# Optimizing Maxillomandibular Position in Orthognathic Surgery: Introducing the T Concept in Treatment Planning

**DOI:** 10.3390/cmtr18040045

**Published:** 2025-10-25

**Authors:** Abdulmalik Alyahya, Saud Bin Jasser

**Affiliations:** 1 Oral and Maxillofacial Surgery Department, Security Forces Hospital, Ministry of Interior, Riyadh 12625, Saudi Arabia; 2 Oral and Maxillofacial Surgery Department, King Abdulaziz Medical City, Ministry of National Guard Health Affairs, Riyadh 11426, Saudi Arabia; saudsmaj@gmail.com

**Keywords:** genioplasty, bimaxillary, orthognathic, surgery, virtual surgical planning

## Abstract

**Background**: Orthognathic surgery aims to align the jaws with the facial skeleton and correct dental occlusion. This paper introduces the concept of planning the maxillomandibular complex (MMC) as a whole, utilizing a t-forming set of landmarks: the maxillary central incisor, the chin, and the occlusal plane. **Methods**: The background, hypothesis, and rationale of the new T concept are explained. A case of a 28-year-old male with skeletal class III malocclusion and an open bite was used to illustrate the application of the T concept in step-by-step surgical planning. The planning encompasses four phases: Phase One involves correcting frontal deformity and various asymmetries, Phase Two involves correcting chin anterior–posterior deformity, Phase Three involves correcting anterior–posterior and vertical MMC position, and Phase Four involves correcting MMC rotation. **Results**: The T concept provided a structured approach to plan MMC as a whole and integrate all structures into harmony. **Conclusions**: The T concept provides a logical approach to MMC positioning in orthognathic surgery, addressing functional and aesthetic concerns. It acts as a checkpoint to verify MMC position, helping surgeons achieve better results and avoid compensatory procedures.

## 1. Introduction

Orthognathic surgery aims to align the maxilla and the mandible to the rest of the facial skeleton and correct dental occlusion [1,2,3]. Although other facial bones may be hypoplastic or deformed in dentofacial deformities, correcting the jaws in harmony with the face would improve the overall aesthetics [1,2,3]. While the parameters of normal occlusion are clear and enjoy unanimous consensus, the parameters of positioning the maxillo-mandibular complex (MMC) in relation to the face have been controversial [4,5,6,7,8,9]. Since the introduction of skeletal cephalometric analysis, different landmarks have been suggested to predict the ideal position of the MMC [4,5,6,7,8,9]. Soon after, there was a paradigm shift from skeletal to soft tissue and clinical landmarks; however, the controversy has not been entirely resolved [10,11,12]. Moreover, the introduction of 3D planning has changed the perspective on which these landmarks were based and initiated another paradigm shift in the concept of proper MMC position [13,14,15,16,17,18,19,20]. This controversy only concerns the sagittal plane, as aligning the midline and addressing frontal asymmetry are not points of disagreement. Surgeons often use their reference lines and angles to position the maxillary central incisor, assuming that if the maxillary central incisor is positioned correctly, the MMC will follow [5,21,22]. While the central incisor is in the correct position, the complex can be rotated either clockwise or counterclockwise, resulting in different facial profiles. Hereby, we introduce the T concept (maxillary central incisor, chin, and occlusal plane) as a new and more practical method for the proper position of MMC as a whole.

## 2. The T Concept

**Figure 1:**
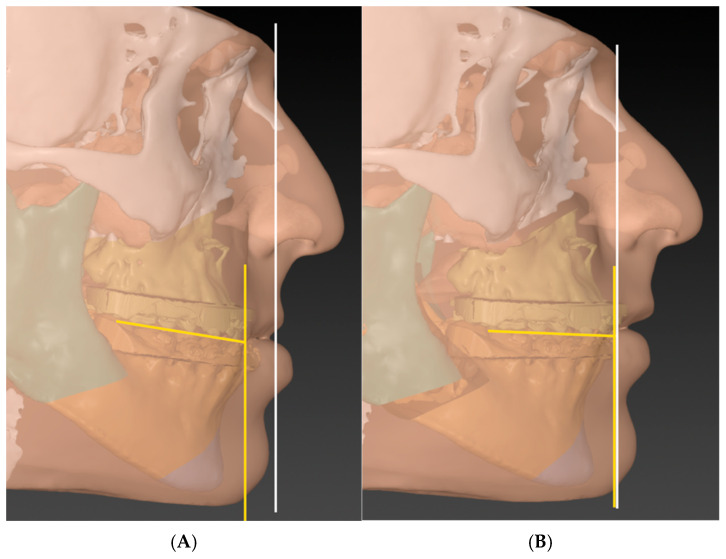
Illustration of the T-concept: (**A**) before and (**B**) after.

The surgical planning literature focused primarily on positioning the maxillary central incisor correctly. The T concept plans the MMC as a whole using the chin and occlusal plane in addition to the central incisors. Landmarks such as vertical lines through soft tissue Glabella, Nasion, or Subnasale were suggested to position the central incisor [5,11]. Based on clinical judgment, the surgeon may choose to place the maxillary incisor on or in front of these lines. The rotation of the MMC is determined by the occlusal plane on one hand and the position of the chin on the other hand. This new concept relies on the “T” formed by the occlusal plane and a vertical line from the labial surface of the maxillary central incisor to the soft tissue gnathion, as illustrated in Figure 1. In an orthodontically well-prepared cases, the head of the T should be aligned with the vertical reference, and the occlusal plane should be within 4–12 degrees to the tail of the T [8,23].

## 3. The Chin Rationale

**Figure 2:**
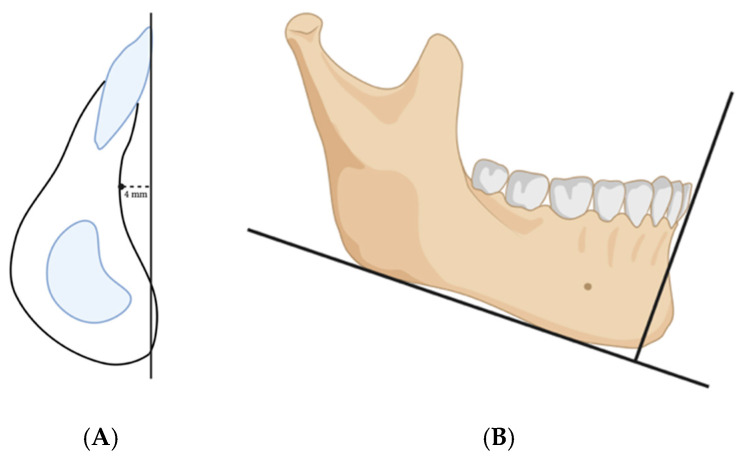
A normal chin–mandible relationship should be assessed before planning the MMC anterior–posterior position. In an orthodontically well-prepared mandible, (**A**) The chin should form a lazy S with pogonion 4 ± 2 mm in front of the B point. (**B**) The chin should be positioned in front of a line perpendicular to the mandibular plane, tangent to the labial surface of the incisors. One must always consider the height of the chin and the soft tissue thickness.

Our hypothesis states that if the chin is in a normal relationship with the mandible preoperatively, correcting the MMC will put the chin in a normal relationship with the face (normal profile). Consequently, if the chin is in a normal relationship with the mandible preoperatively, and after positioning the MMC, the chin becomes out of a normal relationship with the face (i.e., requiring genioplasty), then the MMC position is incorrect (Figure 2).

**Figure 3:**
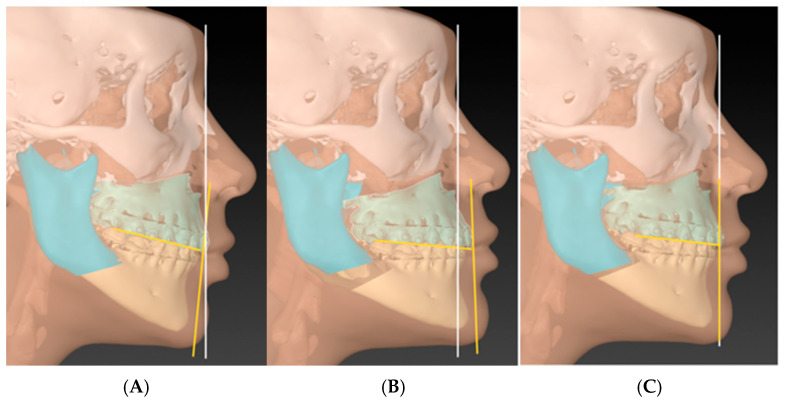
After planning the MMC position, the chin should be evaluated in relation to the skull. (**A**) shows the chin is too far backward due to the clockwise-rotated MMC. (**B**) shows the chin is too far forward due to over-advancement and counterclockwise rotation. (**C**) shows the harmony of the occlusal and mandibular plane with the anterior–posterior position of MMC and the chin. The chin–mandible relation is within normal.

Simply put, when planning a case, one must determine whether the chin–mandible relationship is normal. If the chin–mandible relationship is normal, then planning can proceed as usual. If not, genioplasty is planned to correct the chin–mandible relationship before moving the MMC. Now, once the MMC has been positioned according to the clinical plan, the surgeon will evaluate the position of the chin. If the chin is too far forward, it indicates that the advancement of the MMC is excessive or that the MMC is counterclockwise-rotated. Conversely, if the chin is too far backward, it indicates that the MMC is not advanced enough or that it is clockwise-rotated (Figure 3).

## 4. Step-by-Step Planning

**Figure 4:**
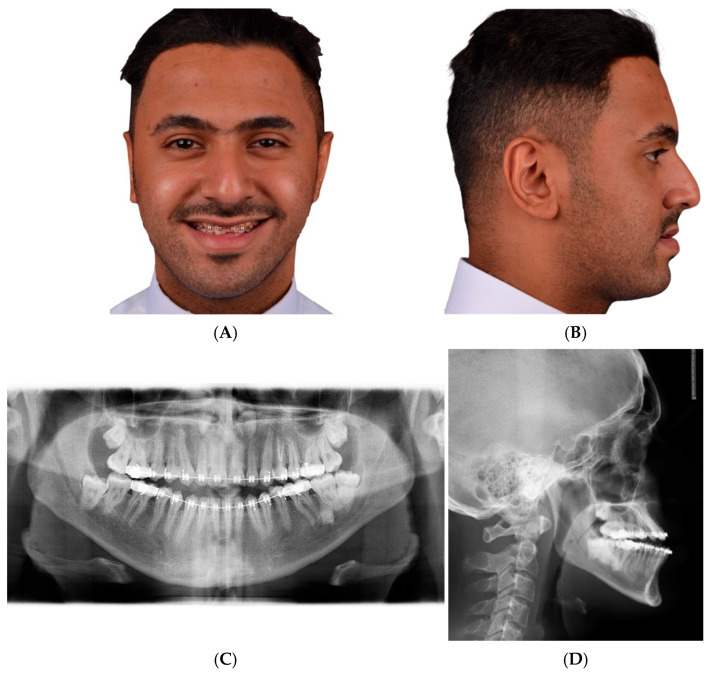
(**A**) Clinical frontal and (**B**) lateral photos, (**C**) panoramic and (**D**) lateral cephalometric radiographs of the patient. The mandible appears relatively prognathic in the lateral cephalometric radiograph. However, the presence of an impacted third molar is an indication that the jaw is shorter than it should be, which means a retrognathic mandible. More importantly, when compared to the clinical photo, it appears obviously misleading.

**Figure 5:**
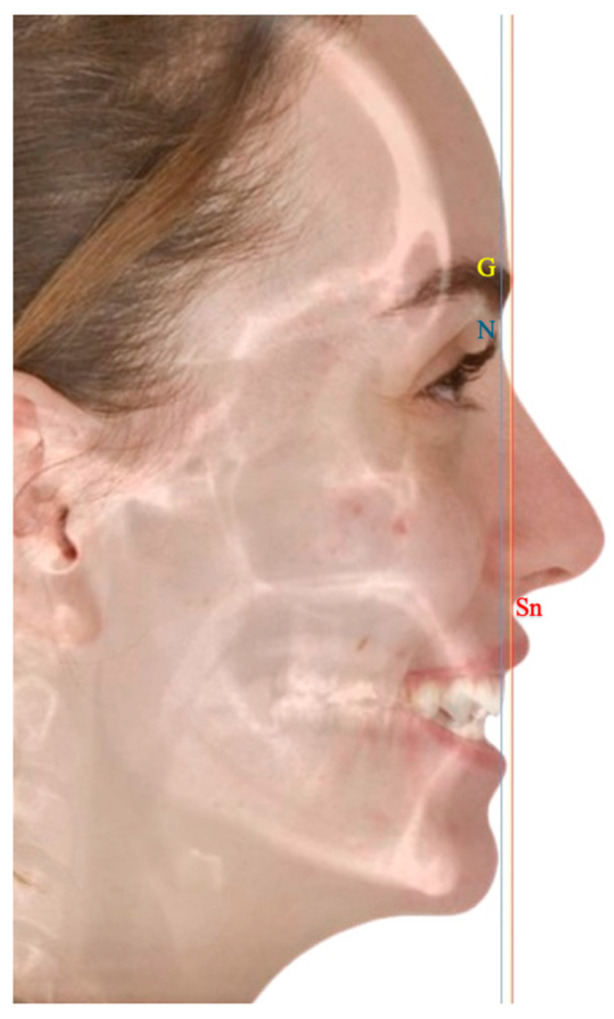
The blue line demonstrates the Barcelona line, defined as a soft tissue nasion (N’) vertical line perpendicular to the natural head position, the yellow line demonstrates the glabellar (Gl) vertical line, and the red line demonstrates the true vertical line passing through subnasale point (Sn). One may notice that these landmarks are very close to each other.

To implement our principle, we present a case with step-by-step planning. Witnessed written and verbal informed consent, granting permission for the use of the patient’s data in publication, was obtained. The case was planned using IPS CaseDesigner^®^ Software V 2.5 (KLS Martin Group, Tuttlingen, Germany) according to Professor Swennen’s published ten steps, with the modification to implement the T concept [24]. A 28-year-old man presented to our clinic complaining of an unpleasant smile and an open bite. The clinical and radiographic findings showed an asymmetry, class III skeletal malocclusion with an open bite, and a broken central incisor. His teeth-show at rest was insufficient. The plan was to correct his asymmetry, advance both jaws, and correct the open bite (Figure 4). The author used a glabella line based on Andrew’s analysis as an advancement landmark; however, he also uses a true vertical line and a Barcelona line, depending on the case’s smile esthetics and overall profile (Figure 5) [5,11,25].

### 4.1. Phase-One

**Figure 6:**
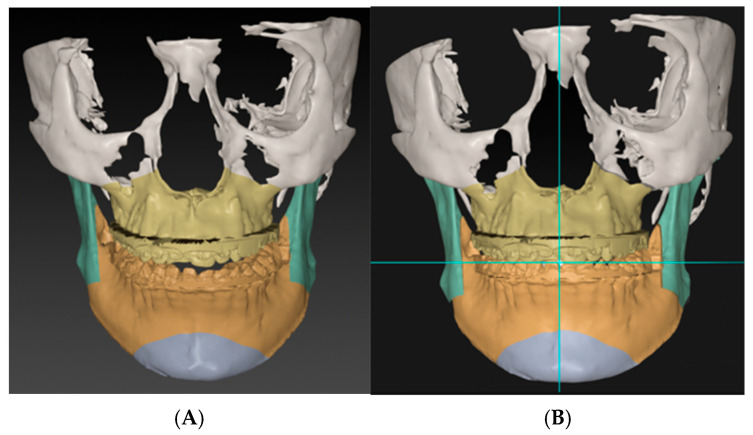
Phase one: As in most virtual surgical planning software, the maxilla and the mandible move together in target occlusion. Going through Swennen’s ten steps, frontal cant, midline, proximal segments, yaw, and chin symmetry are corrected first [24]. (**A**) before correction and (**B**) after.

Planning should begin after acquiring the necessary data, orienting the head, and segmenting the augmented 3D model. The jaws will be locked into the target occlusion, and the plan will be either maxillary-based or mandibular-based. In this phase, frontal asymmetries should be corrected, such as occlusal cant, midline, proximal segment asymmetry, MMC yaw rotation, and chin asymmetry. Following the sequence of these steps is crucial (Figure 6).

### 4.2. Phase-Two

**Figure 7:**
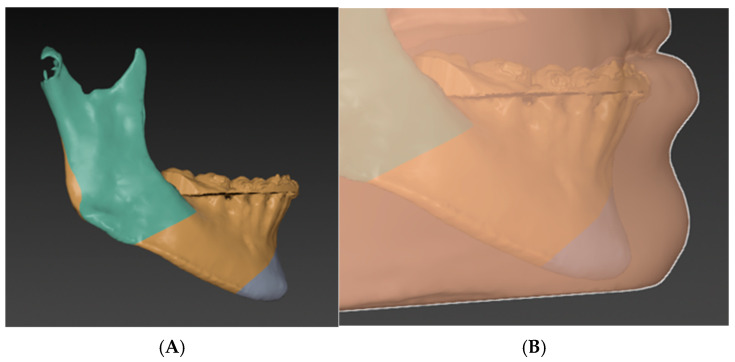
Phase two: (**A**) The chin–mandible relation should be evaluated independently. The decision to do genioplasty or not should be made at this step. (**B**) The soft tissue thickness over the chin should be taken into consideration.

The chin–mandible relationship in lateral view is then evaluated independently from the rest of the facial skeleton. If the chin is deformed either in an anterior–posterior or vertical dimension, genioplasty is planned accordingly. The surgeon should always consider the soft tissue thickness over the chin (Figure 7) [26,27,28,29].

### 4.3. Phase-Three

**Figure 8:**
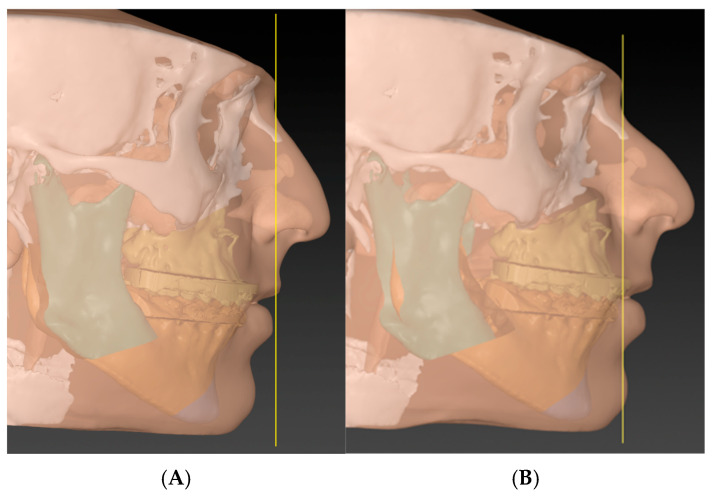
Phase three: Using the maxillary central incisor, the advancement of the MMC is performed based on the surgeon’s preferred vertical reference. Now, the vertical position of the maxillary incisor is adjusted based on the pre-operative incisal show plus the show that is gained by the advancement (roughly every 3 mm of advancement will give 1 mm more show) [30]. In this case, the pre-operative teeth show was 1 mm, and the advancement was 10 mm, so the decision was made to impact 2 mm to leave about 2 mm, considering the broken incisor. (**A**) without advancement reference and (**B**) with glabella line as an advancement landmark.

Now, the surgeon will determine the anterior–posterior position of the MMC based on the vertical reference line and then calculate the resulting teeth-show. We roughly believe that for every 3 mm of maxillary advancement, there will be an additional 1 mm of teeth display [30]. The MMC vertical position is then corrected according to the teeth display. In this step, one should consider the upper lip length and the possible soft tissue response to the planned procedure (e.g., V-Y procedure) (Figure 8).

### 4.4. Phase-Four (T Concept)

**Figure 9:**
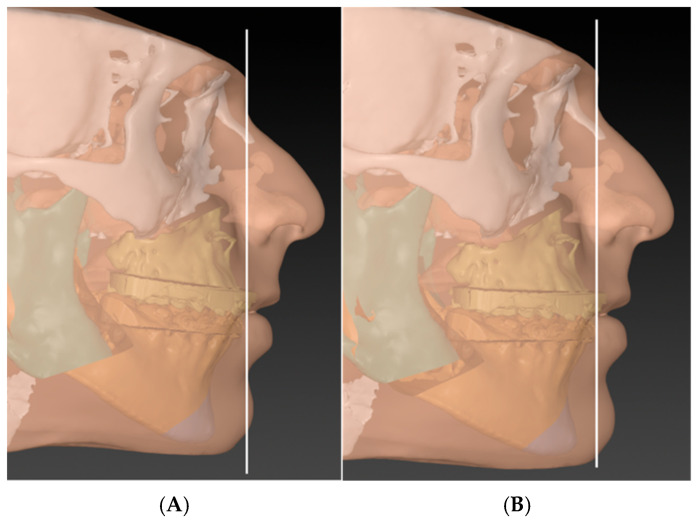
Phase four (the concept step): Instead of rushing towards performing genioplasty to correct the profile, the rotation of the MMC is achieved. The concept is to anchor the central incisor as a rotation point and then move the MMC till the chin is in the proper position (considering the soft tissue thickness). In cases well prepared orthodontically, the amount of rotation needed to put the chin in the correct profile will always match the amount required to correct the mandibular and occlusal planes. (**A**) before MMC rotation and (**B**) after rotation.

**Figure 10:**
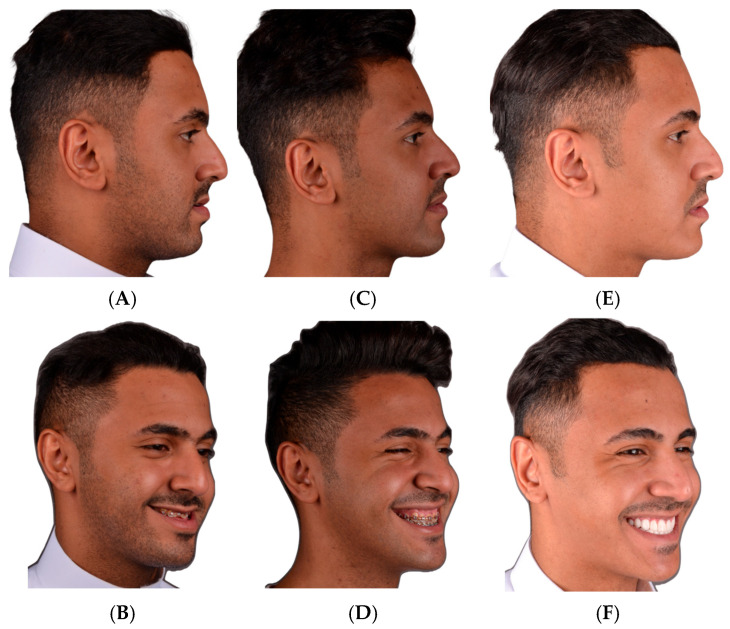
Clinical photographs of the same patient. (**A**,**B**) Pre-operative, (**C**,**D**) 6 months post-operatively, (**E**,**F**) 2 years post-operatively. The net movement of the mandible was advancement.

The surgeon finally rotates the MMC in a sagittal plane, either clockwise or counterclockwise, to level the occlusal and mandibular planes and to align the chin to the facial profile (Figure 9). The clinical results are presented in (Figure 10).

## 5. Discussion

**Figure 11:**
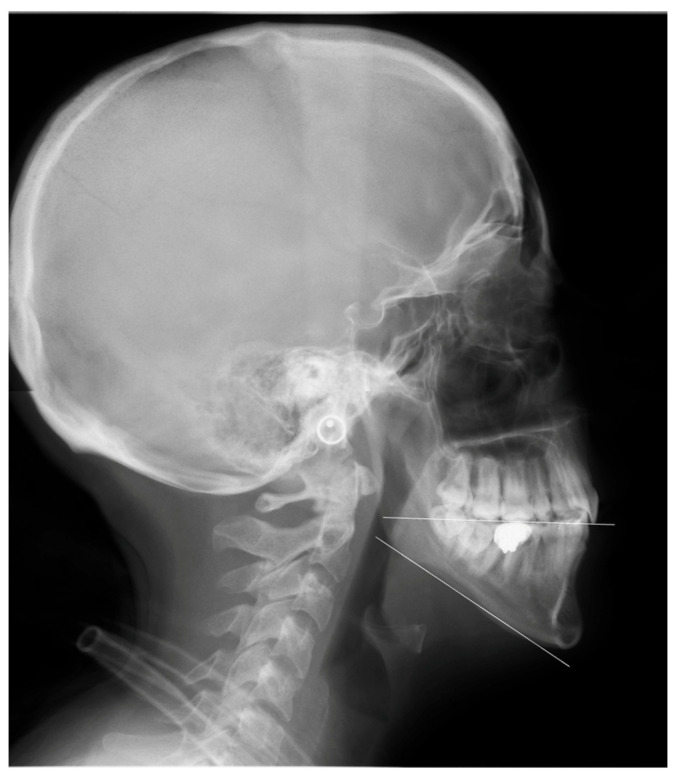
Lateral cephalometric demonstrating a case with an abnormal OP–MP angle, where the expected alignment between the inferior border step and the required MMC rotation cannot be achieved.

The six elements of orofacial harmony, as introduced by Andrews, include central incisor and chin parameters that are evaluated independently of each other [25]. Both should be aligned with the Goal Anterior Limit Line (GALL), which uses the glabella as its reference point [25]. Since the chin and maxilla determine the projection of the face, the occlusal plane is key to defining the rotation of the complex. The rotation of the maxillomandibular complex (previously called rotation of the occlusal plane) was introduced in the late 1980s as an alternative to conventional orthognathic planning and has since become an essential part of it [31,32,33]. The conventional concerns regarding the rotation of the maxillomandibular complex were about the stability of the movement in the presence of powerful musculature, such as the pterygomandibular sling [34]. In the era of three-dimensional virtual planning, as the MMC is moved (within the target occlusion), the inferior border of the distal segment can be evaluated for potential sling impingement [35]. Additionally, the current practice of short splits, Hunsuck modification, makes this potential risk even lower [36,37]. In our experience, the planned advancement and vertical repositioning of the MMC (phase three) will create an inferior border step that perfectly matches the necessary rotation of the MMC. This might not be true in cases where the Occlusal Plane–Mandibular Plane angle (OP-MP Angle) is not normal (Figure 11). The proximal segment can also tolerate minor pitch rotation depending on its preoperative deformity.

Although the planning phases described in this article address all deformity dimensions, the T-concept is limited to the lateral view planning. From a frontal view, the primary planning objective is symmetry, making it easy to relate structures from one side to another (phase one). However, in the lateral view, we depend on the linear and angular relationships between the skull and the MMC. The position of the MMC cannot be precisely determined using just one point, i.e., the central incisor. There are norms for maxilla, mandible, chin, occlusal plane, and so on, but there are no norms for the MMC as a whole. Since pre-surgical orthodontics decompensates dentoalveolar deformities and establishes a normal jaw-to-teeth relationship, when the maxilla and mandible are brought into occlusion, they will function as a harmonious unit. Now, this unit from the lateral aspect forms a T, where the maxillary incisors and the chin form the head of the T, and the occlusal plane forms the tail. When the maxillary incisor is aligned with the reference vertical line, the chin should be within 4 mm of that line, and the occlusal plane should be angled 4 to 12 degrees from a perpendicular line to it [5,8]. We chose gnathion as the chin landmark to account for the soft tissue thickness of the chin.

**Figure 12:**
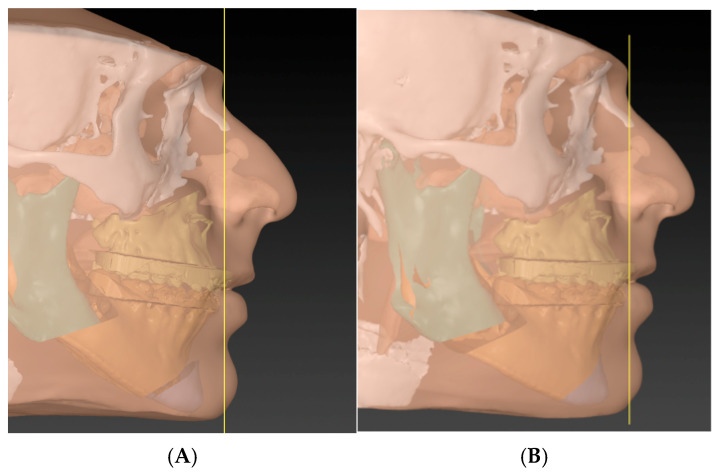
Comparison between traditional planning (**A**) and the T concept (**B**) demonstrating that, in traditional planning, an additional genioplasty is required to achieve a similar facial profile, whereas the T concept attains the desired harmony through MMC repositioning alone.

This concept faces a challenging situation because genioplasty may disrupt the supposed MMC harmonious unit. Furthermore, the surgeon’s subjectivity is more evident when it comes to genioplasty. A recent study by Palla et al. examined the differences among experienced surgeons in planning orthognathic procedures. The largest deviation, in both the vertical and anterior–posterior directions, was found at the pogonion point [38]. In clinical practice, the decision regarding genioplasty is often deferred until after MMC planning. When the MMC is insufficiently advanced or improperly rotated clockwise, an advancement genioplasty is needed to restore the facial profile (Figure 12). This raises the question: Should such a procedure be considered a camouflage genioplasty? By definition, camouflage genioplasty masks an underlying jaw deformity [39,40,41]. If the skeletal discrepancy has already been corrected through bimaxillary surgery, genioplasty should only address the deformity of the chin itself. Therefore, it is wise to evaluate the chin deformity and decide whether genioplasty is necessary prior to MMC planning (phase two). Once its relationship with the mandible is established, the chin can serve in the T-complex as a reliable guide for determining the optimal position and rotation of the MMC. This, in theory, is almost similar to the decompensation of the teeth prior to orthognathic surgery. It delineates the actual deformity of the jaws and makes planning more accurate and predictable. This concept introduces a new philosophy in surgical planning, which we call “visual planning.” Instead of converting the patient’s deformity into numerical values and using these to adjust the MMC in the virtual model, we directly correct the deformity on the patient’s model in real time using the T concept. It is similar to aligning a picture frame on a wall by visually estimating its position relative to the floor. Since this approach relates the T complex to surrounding facial structures, its applicability may be limited in syndromic or severe craniofacial deformities.

## 6. Conclusions

In contemporary orthognathic surgery, virtual planning provides a significant advantage by enabling real-time visualization of the movement of all structures of the MMC during the planning process. This may not be the first report to recommend adopting MMC planning as a whole; however, it demonstrates how well-decompensated jaws and other structures can be brought into harmonious norms. It is essential to establish the MMC unit by planning the chin first, rather than leaving it to the last minute to compensate for MMC planning shortcomings. Since we adopted this method, the rate of genioplasty has not exceeded 30% of our bimaxillary orthognathic cases. We believe that most cases of dentofacial deformities have a normal chin–mandible relation, and if there is a sagittal chin deformity, advancements of a few millimeters are often sufficient to correct it. Surgeons should reconsider their surgical planning if they consistently find the need for genioplasty in most cases or require significant advancements in genioplasty. This will ensure that genioplasties are not just masking insufficient advancement or rotation of the MMC. The only exception is functional genioplasty in cases of OSA and orthodontically compromised cases, where genioplasty is considered a camouflage.

## Data Availability

All available data are within the manuscript.

## Abbreviations

The following abbreviations are used in this manuscript:

MMC	Maxillomandibular complex
GALL	Goal Anterior Limit Line
OP-MP Angle	Occlusal Plane–Mandibular Plane angle
